# Collaboration for implementation of decentralisation policy of multi drug-resistant tuberculosis services in Zambia

**DOI:** 10.1186/s12961-024-01194-8

**Published:** 2024-08-19

**Authors:** Malizgani Paul Chavula, Tulani Francis L. Matenga, Patricia Maritim, Margarate N. Munakampe, Batuli Habib, Namakando Liusha, Jeremiah Banda, Ntazana N. Sinyangwe, Hikabasa Halwiindi, Chris Mweemba, Angel Mubanga, Patrick Kaonga, Mwimba Chewe, Henry Phiri, Joseph Mumba Zulu

**Affiliations:** 1 Department of Community and Family Medicine, School of Public Health, University of Zambia, Lusaka, Zambia; 2 Department of Epidemiology and Global Health, Umeå University, 901 87 Umeå, Sweden; 3 Department of Health Promotion and Education, School of Public Health, University of Zambia, Lusaka, Zambia; 4 Department of Health Policy and Management, School of Public Health, University of Zambia, Lusaka, Zambia; 5 Yakini Health Research Institute, Lusaka, Zambia; 6 Ministry of Health, Kitwe Teaching Hospital, Off Kumboka Drive, P.O. Box 20969, Kitwe, Zambia; 7 Department of Epidemiology and Biostatistics, School of Public Health, University of Zambia, Lusaka, Zambia; 8 Department of Environmental Health, School of Public Health, University of Zambia, Lusaka, Zambia; 9 Ministry of Health, Ndeke House, Haile Selassie Avenue, P.O. box 30205, Lusaka, Zambia

**Keywords:** Collaboration, collaborative governance, principled engagement, shared motivation, capacity for joint action, decentralisation, policy, MDR-TB, system context

## Abstract

**Background:**

Multi-drug-resistant tuberculosis (MDR-TB) infections are a public health concern. Since 2017, the Ministry of Health (MoH) in Zambia, in collaboration with its partners, has been implementing decentralised MDR-TB services to address the limited community access to treatment. This study sought to explore the role of collaboration in the implementation of decentralised multi drug-resistant tuberculosis services in Zambia.

**Methods:**

A qualitative case study design was conducted in selected provinces in Zambia using in-depth and key informant interviews as data collection methods. We conducted a total of 112 interviews involving 18 healthcare workers, 17 community health workers, 32 patients and 21 caregivers in healthcare facilities located in 10 selected districts. Additionally, 24 key informant interviews were conducted with healthcare workers managers at facility, district, provincial, and national-levels. Thematic analysis was employed guided by the Integrative Framework for Collaborative Governance.

**Findings:**

The principled engagement was shaped by the global health agenda/summit meeting influence on the decentralisation of TB, engagement of stakeholders to initiate decentralisation, a supportive policy environment for the decentralisation process and guidelines and quarterly clinical expert committee meetings. The factors that influenced the shared motivation for the introduction of MDR-TB decentralisation included actors having a common understanding, limited access to health facilities and emergency transport services, a shared understanding of challenges in providing optimal patient monitoring and review and their appreciation of the value of evidence-based decision-making in the implementation of MDR- TB decentralisation. The capacity for joint action strategies included MoH initiating strategic partnerships in enhancing MDR-TB decentralisation, the role of leadership in organising training of healthcare workers and of multidisciplinary teams, inadequate coordination, supervision and monitoring of laboratory services and joint action in health infrastructural rehabilitation.

**Conclusions:**

Principled engagement facilitated the involvement of various stakeholders, the dissemination of relevant policies and guidelines and regular quarterly meetings of clinical expert committees to ensure ongoing support and guidance. A shared motivation among actors was underpinned by a common understanding of the barriers faced while implementing decentralisation efforts. The capacity for joint action was demonstrated through several key strategies, however, challenges such as inadequate coordination, supervision and monitoring of laboratory services, as well as the need for collaborative efforts in health infrastructural rehabilitation were observed. Overall, collaboration has facilitated the creation of a more responsive and comprehensive TB care system, addressing the critical needs of patients and improving health outcomes.

## Introduction

Multi-drug-resistant tuberculosis (MDR-TB) infection  is a major global public health concern, with TB remaining as one of the top 10 leading causes of morbidity and mortality, especially in low- and middle-income countries (LMICs) [1]. In 2022, the global MDR-TB burden estimate was at 410 000 cases (CI 370 000–450 000) and only 176 000 (43%) were initiated on treatment [2]. The burden of MDR-TB infection and disease is unevenly distributed globally, with LMICs disproportionally affected due to high poverty levels [1]. Zambia is among 30 other countries with the highest MDR-TB burden in the world [1]. In 2022, Zambia had an estimated burden of 1900 MDR-TB cases, but only initiated treatment in 362 cases in the same year (WHO 2023 Global TB Report). The country recorded a treatment success rate for MDR-TB of 79% for the same year, which was lower than the treatment success rate for drug-susceptible TB, which was at 92%. The sub-optimal treatment success for MDR-TB cases is attributed to the complexity of the TB bacterium called *Mycobacterium tuberculosis,* as it undergoes mutations, rendering it resistant to first-line drugs crucial for TB treatment, hence requiring a more comprehensive and multifaceted approach during treatment and care [3].

Studies have highlighted risks and susceptibility factors, which drive MDR-TB infection. These include gender, residence, history of previous TB treatment, lack of knowledge and poor adherence to treatment, treatment failure, presence of MDR-TB in the family and low economic status [5, 6]. Further, treatment success is hindered by adverse events that may arise during treatment, including vomiting, skin rash, anaemia and peripheral neuropathy [7]. Drivers for unsuccessful treatment outcomes include social stigma, negative experiences of physical and emotional trauma, lack of social support and non-responsiveness to healthcare services [8]. Therefore, while MDR-TB is driven by various factors such as gender and social support, its successful treatment faces challenges from both side effects and patient experiences.

Prevention of MDR-TB infection is part of the global agenda of Sustainable Development Goal (SDG) 3 (Good Health and Well-being), thus, in practical terms, the aim is to dismantle inequalities and increase universal health coverage [9]. Many countries are adopting decentralisation of MDR-TB services through health systems strengthening as a critical way of ensuring timely service delivery to all people. Global partners and international organisations are playing a critical role in strengthening the health systems through resource mobilisation, and investment into improving infrastructure, diagnostics, health information and human resources for health development, to enhance service delivery [10].

Studies have revealed that decentralisation of MDR-TB healthcare services has had significant advantages including improved accessibility, and timely delivery of care particularly for rural areas [11]. In Bangladesh, decentralisation contributed to enhanced collaboration in localising MDR-TB medical services, adapting them to local preferences and needs [12]. However, governance issues such as fragmentation and poor coordination remain significant gaps limiting equitable resource distribution for MDR-TB services, including infrastructure inadequacy. Many other challenges, however, are faced by many countries in trying to combat TB and attain the WHO global target to eliminate TB by 2030, through the End TB Strategy [10]. In South Africa, healthcare providers reported anxiety over the abrupt introduction of MDR-TB care, limited support and inadequate communication and collaboration during the service implementation [7]. These challenges are exacerbated by socio-economic and political factors including declining funding towards TB services .

In 2017, Zambia’s Ministry of Health introduced a policy to decentralise MDR-TB services through the 2017–2021 National Strategic Plan for Tuberculosis and Leprosy Prevention, Care, and Control, which was aligned to the National Health Strategic Plan and the WHO Global End TB Strategy [13]. The MDR-TB service delivery has, since 2017, been decentralised from the two national-level hospitals to about 100 sites across all 10 provinces in the country, including regional and local hospitals. The Ministry of Health has been collaborating with local and international organisations to support the delivery of decentralised TB services. Some of the funding agencies working with the Ministry of Health in supporting the decentralisation process include the Global Fund, the United States Government through  the United States Agency for International Development (USAID) and Centers for Disease Control and Prevention (CDC), WHO, Japan Anti-Tuberculosis Association (JATA) and many others. Local partners such as civil society organisations (CSOs), TB survivor groups, faith-based organisations and many others have also been key in enhancing the decentralisation process in the country. In line with this strategic direction, collaboration has the potential to create an opportunity to strengthen the health system through increasing coverage, expanding access and improving the comprehensive availability of MDR-TB services across the country.

Collaboration is a participatory process of engaging key actors in addressing complex problems that cannot be handled by a single entity. Some studies have been conducted in LMICs on collaborative governance of tuberculosis control programmes (West Africa and Bangladesh) [14, 15]. The Ministry of Health in Zambia, in collaboration with partners, is implementing the decentralisation of MDR-TB services. There is inadequate evidence on the optimal implementation of decentralised MDR services in the country with available literature only focusing on the general TB and human immunodeficiency virus (HIV) programme collaborative activities [16]. Most studies conducted have not addressed how system context issues and capacity for joint action as aspects of collaboration affect the effective or successful decentralisation of MDR-TB services. This study sought to explore the role of collaboration in the implementation of the decentralisation policy of multi-drug-resistant tuberculosis services in Zambia.

### Conceptual framework: integrative collaborative governance

**Fig. 1:**
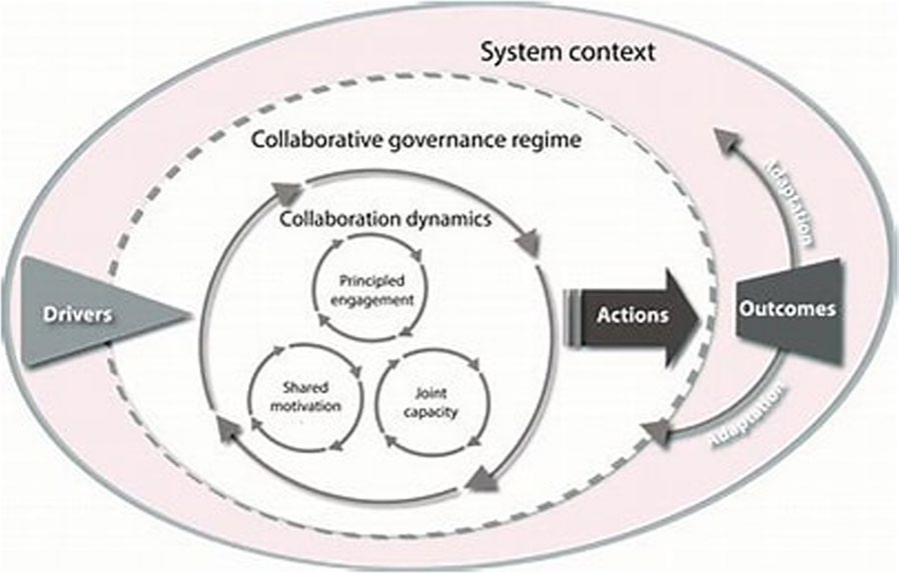
Integrated framework for collaborative governance Emerson et al. [17]

To address the research question, we adopted an integrated framework for collaborative governance to analyse the findings according to Emerson et al. [17]. Collaborative governance is defined as “the processes and structures of public policy decision-making, and management that engage people constructively across the boundaries of public agencies, levels of government, and/or the public, private and civic spheres to carry out a public purpose that could not otherwise be accomplished” [5]. We adopted the integrative framework for collaborative governance by Emerson et al. [17] to analyse the role of collaboration in the implementation of the decentralisation policy of multi-drug-resistant tuberculosis services in Zambia. The framework consists of key components (layers) including system context, collaborative governance regime, drivers and collaborative dynamics (principled engagement, shared motivation and capacity for joint action) [2] as shown in Fig. 1. However, this paper focussed on exploring how collaboration dynamics namely principled engagement, shared motivation and capacity for joint action c hinder or support the implementation decentralisation policy of MDR-TB services in Zambia. The interaction and intersectionality of contextual actors including the political, social and legal environment are some of the key drivers influencing collaboration dynamics. The concept of principled engagement entails a process that unfolds over time, involving various stakeholders who may participate at different stages and in different settings, such as face-to-face or virtual meetings, cross-organisational networks or public and private gatherings. In this study, stakeholders engage through principled discussion to define the purpose, guidelines and roles necessary to govern the collaboration. The degree of shared motivation among actors influences the nature and pattern of collaboration in the delivery of MDR-TB services. Furthermore, capacity for joint action refers to the actor’s ability to collectively decentralise the delivery of MDR-TB services. The stakeholders collectively, through regular joint meetings, mobilise resources to facilitate implementation of MDR-TB services using existing networks and community structures [3].

## Methods

### Study context

This study was conducted in selected health facilities in Zambia, where the burden of TB, particularly MDR-TB, is high. The contributing factors to the higher prevalence include poverty, rapid urbanisation, population growth and exposure to silica in mining settlements [17]. In response to this situation, the Ministry of Health (Zambia), in collaboration with partners, implemented the decentralised treatment and management of TB from two national health facilities (in Lusaka and Ndola) to other facilities in all 10 provinces. The decentralisation of TB services was implemented in alignment with the 2022–2026 Zambia National Health Strategic Plan for Tuberculosis, which stresses the significance of adopting the primary healthcare approach in eliminating MDR-TB by 2030 [12]. The study was conducted in various selected healthcare facilities, including provincial and district hospitals, both public and private across the nation (Lusaka, eastern, southern, western, central and Copperbelt provinces). The study sites were selected on the basis of their higher volumes of MDR-TB case notifications, with decentralisation of TB services already being implemented in these sites.

### Study design

A qualitative case study design was adopted to investigate the influence of collaboration on decentralising drug-resistant tuberculosis services in Zambia. The application of this approach enabled a comprehensive analysis of the collaboration in the implementation process. We used a case study approach to get a detailed understanding of the collaboration within the context of MDR-TB. Case studies are useful when conducting a detailed exploration of an issue in its real-life context, such as collaboration in the implementation of MDR-TB, and was relevant to facilitate unpacking of substantive real-life contexts, interactions and complexities [18]. The study utilised this design to understand how collaboration influenced the success and challenges of the decentralisation process.

### Data collection methods and sampling strategy

In this study, we employed key informant and in-depth interviews as methods of understanding collaboration for the implementation of decentralisation policy of multi-drug-resistant tuberculosis services in Zambia. We conducted a total of 112 interviews with healthcare workers (18), community health workers (17), patients (32) and caregivers (21) in select healthcare facilities located in 10 selected districts and key informant interviews with facility, district, provincial, and nationallevel based managers (24). We engaged 10 trained research assistants who conducted various study activities under the supervision of the study team. The research assistants were divided into groups and collected data from the different facilities. Study participants were purposively sampled based  on their involvement in the treatment and management of TB at different levels. Table 1 summarises the qualitative interviews per category of respondents.

**Table 1:** Participant information

Interview categories	Subtotal
Healthcare workers	18
Community health workers	17
Patients	32
Caregivers	21
Key informant interviews managers (facility, district, provincial and national levels)	24
Total	112

### Data management and analysis

The collected interviews were transcribed word for word and managed using NVivo software plus 14. We adopted an integrative collaborative governance framework focussing on collaboration dynamics to guide the analysis. A codebook was developed in NVivo and trained research assistants then used the NVivo software and coded the transcripts on the basis of the pre-determined coding framework. Subsequently, the coded projects were integrated into a unified project. The coding process enabled us to identify codes, which were later grouped into substantive themes. These substantive themes were later aligned with the respective domains under collaboration dynamics including principled engagement, shared motivation and capacity for joint action [19]. Our analysis approach was guided by the thematic data analysis method [19].

### Trustworthiness of the study

To ensure the credibility and trustworthiness of the study, transcripts were coded by different coders. After coding, the authors verified the coded work to ensure that the quotes were representative of the developed codes. Additionally, quality assurance of transcripts was conducted through the sharing of transcripts with study team members and audio recordings. Furthermore, we held meetings with stakeholders who participated in the study to discuss the findings. However, this did  not affect the interpretation of the themes as participants confirmed or could relate to these findings.

### Findings

This section presents collaboration dynamics strategies shaping the implementation of the decentralisation policy of MDR-TB services. The results have been presented around the integrative collaborative governance domains, including principled engagement, shared motivation and capacity joint action, as highlighted in Table 2 below.

**Table 2:** Collaboration for implementation of decentralization policy of multi-drug-resistant tuberculosis services in implementation in Zambia

Domains	Subthemes
Principled engagement	• Global health agenda/summit meeting influence on decentralisation of TB • Political will to support introduction of decentralisation • Engagement of stakeholders to initiate decentralisation • Supportive policy environment for the decentralisation process • Quarterly clinical expert committee meetings
Shared motivation	• Common understanding: limited access to health facilities and emergency transport services • Shared understanding of challenges in providing optimal patient monitoring and review • Appreciation of the value evidence-based decision-making in adopting of MDR- TB decentralisation
Capacity for joint action	• Leadership capacity in communicating the implementation plan • MoH initiating strategic partnerships in enhancing MDR-TB decentralisation • Leadership capacity in organising the training of healthcare workers • Training of multidisciplinary teams • Inadequate coordination, supervision and monitoring of laboratory services • Joint action in health infrastructural rehabilitation

### Domain 1: Principled engagement

Principled engagement was shaped by the global health agenda/summit meeting influence on decentralisation of TB, political will to support the introduction of decentilisation,  engagement of stakeholders to initiate decentralisation, and a supportive policy environment for decentralisation of MDR-TB services. 

### Global health agenda/summit meeting influence on decentralisation of TB

The local government leadership interaction with the global community on health reignited the desire to create systems that increase access to health. Participants narrated that the global meeting on health for all heads of state on sustainable development was held. Goal number three was appreciated by heads of state, including the  available leadership at the time. The notion of decentralising health governance, including the delivery of services, was adopted as part of the government’s agenda. The Zambian Government also committed itself to urgently address gaps in access to TB services. The Ministry of Health was tasked with finding mechanisms to address TB access-related challenges.

*[In] 2015 there was a high-level meeting where heads of state were called at the UN summit and subscribed to the sustainable development goal number three and malaria, TB and HIV were picked globally for contributing as causes of mortalities, so the summit recognised the need to do something about it…* (KII, government official 1).

### Political will to support the introduction of decentralisation

The documented challenges on centralisation received government support, and this was a catalyst for decentralisation of TB services in Zambia. Some participants noted that there was a great push from the Ministry of Health that played a crucial role in preparing for decentralisation. Furthermore, the political will and ownership of appreciation of the value of decentralisation was also enhanced by the global agenda on health where the fight against TB was one of the priorities.

*The government, through the Ministry of Health, emphasises zero cost on the part of the patient who has come to access TB services. There's caution to make sure that patient incur zero (or minimal) cost. So, when we look at these things and certainly say, how can we stop someone from travelling from [the provincial capital] all the way to UTH to seek treatment?* (KII, TB government official 2).

### Engagement of stakeholders to initiate decentralisation

The Ministry conducted capacity building to secure stakeholder buy-in for decentralisation, fostering community support and promoting integration, organisational capacity building, staff recruitment maintenance and ensuring a fertile climate for community support. Respondents indicated that obtaining explicit buy-in from critical stakeholders was necessary to foster a supportive environment through community sensitisation and capacity-building. Partnerships between the Ministry of Health and some implementing partners including local NGOs were essential to enhancing the provision of resources such as funding, equipment and training.

*We built capacities, then we also conducted a lot of sensitisations, in promoting decentralisation, amongst other healthcare workers as well as amongst the patients, we assured the patients that service flow would continue smoothly, they shouldn’t be worried about those people who would be attending to them. They are capable* (KII, government official 3).

Furthermore, organisational capacity was conducted to enhance institutional and structural health systems governance, and overall abilities to deliver quality services effectively and efficiently. Organisational capacity was conducted through recruiting and training new staff, equipping staff, improving infrastructure and increasing access to resources. As one interviewee stated:

*So, now we actually started ah… are equipping, doing capacity-building to health workers in these other facilities which highlights the importance of investing in the development of human resources to improve the overall capacity of the healthcare system* (KII, government official 4).

### Supportive policy environment for the decentralisation process

The government, through the Ministry of Health, introduced policies including the 2017–2021 Zambia National Strategic Plan (NSP) on TB and Leprosy Management and Control in Zambia. To this effect, the Ministry of Health introduced the MDR decentralisation across the provinces in a phased approach. The services were decentralised first in Lusaka and the Copperbelt, and subsequently to other provinces including the Eastern, Western, North-western and Central provinces. However, little was mentioned about the impact that these policies had on operations at various levels.

*There is a strategic document that we have called national strategic document for TB so that once again gives the overall guidance, and it runs for a period of 5 years so that is the mother document. The implementation part is the guideline, where everything is well documented and even algorithms are an extract from the guideline. Even when you go to the lab it will tell you an algorithm to use* (KII, government official 5).

However, interviewees were of the view that the lack of stakeholder involvement during the decentralisation process may have contributed to the removal of critical policy and program features required for the successful implementation of the MDR-TB programme. They felt that engaging stakeholders, particularly healthcare practitioners, would assist them grasp the programme’s importance, build appreciation and allow for talks about how to incorporate the program into their daily activities. The absence of stakeholder participation in these talks may have resulted in missed opportunities.

*I observed the relaxed support to decentralisation program by the district leadership, when you go to the district to do mentorship, our expectation was that the district leadership in most cases were supposed to be with us and just maybe even just participate for 10 minutes, even see what’s happening and have a word with a local team, but in most districts we did not see that, so this resulted in health workers not taking the program to be serious because health workers take the program to be serious when they see the top leadership is also involved* (KII, government official 6).

### Quarterly clinical expert committee meetings

Strengthened healthcare providers’ collaboration was recognised as a strategic approach to improving MDR-TB healthcare reform that could lead to improved patient outcomes. Expert committees were present at national, provincial and district levels. Peer-to-peer data reviews in the districts were felt to be effective. However, the capacity of provincial expert committees to go around districts providing technical assistance and facilitation for the implementation of decentralised services was reliant on the available services such as diagnosis and screening. This has led to a reduced number of visits in the last few years. The TB experts gave midweekly reviews of the performance of the decentralised MDR-TB services and identified strategies to improve them. Clinical expert committee meetings at national and provincial levels were held quarterly to review difficult cases and technical support provided on the best patient management strategies.

*We also hold the quarterly clinical expert committee meetings where we review difficult TB cases pertaining to patients. Each district was given a chance to make a presentation on difficult cases that they have had in that quarter both for MDR and drug susceptibility so in that platform we build capacity, and we have a team of experts that now advise on how that patient can be managed and we have really improved in the treatment outcome for DR patients* (KII, government official 7).

### Domain 2: Shared motivation

Several factors influenced shared motivation in the decentralisation of MDR-TB, including actors having a common understanding, limited access to health facilities and emergency transport services, shared understanding of challenges in providing optimal patient monitoring and review, and their appreciation of the value of evidence-based decision-making in adopting the MDR-TB decentralisation.

### A common understanding of the challenges faced by MDR-TB: limited access to health facilities and emergency transport services

The centralisation of TB services brought about patient discontentment regarding poor service delivery due to the poor accessibility of TB services. The patients were required to travel long distances to selected health facilities for treatment. Some patients with inadequate financial resources could not afford transportation fees to health facilities, accommodation and food while seeking care at the health facilities. The challenges contributed to socio-economic inequalities concerning access to health services. The respondents narrated that there was a great need for the government to adequately deliver these services, especially in provinces such as Eastern and North-western provinces where the decentralisation process was happening at a slow pace and had patients that still experienced difficulties travelling to health facilities.

*I stayed in Lusaka for treatment for 5 months, the sixth month they said the remaining 1 month you should go and finish from home. So, when I came back home the medicine I got from Lusaka was not here the whole week and in the second week I found the medicine, and it happened that the cough came back again. When I thought of coming back to the clinic, I had no transport because where I live........ there is a distance* (Patient with TB).

Centralisation also affected the emergency transport services as more patients were required to be taken to only two facilities in the country. Hence, before decentralisation, health facilities experienced challenges in referring patients. Sometimes, the unavailability of ambulances or transport limited the capacity of health facilities to deliver services to patients in time. During the decentralisation phase, more patients were attended to promptly because several health facilities across districts were offering services to patients with MDR-TB.

*Before decentralisation, so, first a case could be identified by facility, and the facility would communicate to the district, then the district needed to find transport to take that patient to the central treatment centre, yet the district does not have any capacity to transport that patient* (KII, government official 8).

The adoption of decentralisation facilitates opportunities for local health systems to collaborate with existing partners to provide emergency services to the nearest hospital. Compared with taking the patients to the two national treatment centres, the decentralised model reduces costs such as travel costs which were associated with TB management/services before decentralisation.

*I can mention here that for us, we can’t afford a vehicle to go and pick up a client from a facility to the general hospital so our partners will provide the vehicle to move the patient and even if we want to go and visit a patient, our partners will provide transport/logistics* (KII, government official 9).

### Shared understanding of challenges in providing optimal patient monitoring and review

The centralisation of MDR-TB services was perceived to be affecting the monitoring and care of patients. Healthcare workers in the centralised system experienced heavy workloads due to huge numbers of patients, thus making the monitoring of patients challenging and sometimes impossible. Furthermore, seeing many patients and managing patient health information was problematic, furthering the gap in ensuring that patients are effectively monitored. The government and implementing actors recognised the multifaceted challenges and supported the decentralisation process to contribute to a reduction of the problem.

*Patient overload, distance to the facility, poor record keeping and follow-ups were not being done and maybe even monitoring of these patients was difficult, so they figured out that if we decentralise maybe things will be done more orderly. So even patient care was compromised, so when they decentralised care and treatment improved because services were brought closer to home* (KII, government official 10).

### Appreciation of the value of evidence-based decision-making in adopting MDR-TB decentralisation

The capacity readiness assessment included evaluating the size and composition of health facilities, the availability of human resources, diagnostic and laboratory capabilities and the availability of data collection tools. These facilitated an understanding of facilities’ readiness to implement and manage MDR-TB treatment at the facility level. Key informants narrated that human resource for health were identified as a crucial factor, and facilities needed to have at least one medical doctor and a dedicated clinician or nurse trained in DR-TB management to handle the patients. Diagnostic services also had to be available to make an accurate diagnosis of MDR-TB. The decentralisation process was gradual, starting with larger hospitals in 2014 and fully decentralising to districts in 2018. There was also an imperative need for adequate drug stocks, which were crucial in ensuring that facilities could continue providing treatment and care for patients with MDR-TB. The success of the decentralisation process of TB services depended heavily on these preparatory measures, with manpower development being a key factor as one interviewee stated:

*So, we did have a tool that was assessing certain things that should be in place for a site to be set to be related to start treating patients. It has to be a diagnostic site, it must have a preferred medical officer who’s also trained in drug-resistant TB* (KII, government official 11).

### Domain 3: Capacity for joint action

The capacity for joint action strategies included leadership roles in communicating the implementation plan, MoH initiating strategic partnerships in enhancing MDR-TB decentraliation, leadership capacity role in organiing training for healthcare workers, training of multidisciplinary teams, inadequate coordination, supervision and monitoring of laboratory services and joint action in health infrastructural rehabilitation.

### Leadership capacity in communicating the implementation plan

The selected sites were assessed using a tool to ensure that each region had the necessary resources to treat patients with MDR-TB. This strategy allowed for a targeted and context-specific approach to implementing decentralised MDR-TB treatment in Zambia, rather than a one-size-fits-all plan. The communicating of the plan to all relevant implementing partners was crucial to ensuring that they were all informed and guided. Another KII participant stated:

*We have to have different strategies for different provinces because the capacity of one province is not the same as the capacity of another province* (KII, government official 12)

*I think one last important area where we are involved is to make sure that the community TB program is also supported and coordinated so that as a province, we do make sure that drug-resistant TB at the community level is implemented, where volunteers are supported. …. provide services on DRTB by for instance supporting DRTB patients at the community level. (…) even giving education at the community level for people who are coughing or people who may be on treatment but they are not getting any better so communities are involved, so in a nutshell that’s what I can say the degree to which am involved in DRTB program* (KII, government official 17).

### MoH initiating strategic partnerships in enhancing MDR-TB decentralisation

Strategic partner identification was critical to the successful execution of the MDR-TB decentralisation strategy. As a result, several partners were identified to assist with staffing specific facilities, sourcing equipment and providing assistance at the district or facility levels. It has been stated that increased collaboration in healthcare is a strategic approach to reform that can improve patient outcomes, such as reducing preventable adverse drug reactions, lowering morbidity and mortality rates and optimising pharmaceutical dosages.

*The Ministry of Health alone cannot manage to sufficiently do a lot of things [on its own] but when you collaborate with other organisations, it helps because for example, the training which we have been having, they were supported by CIDRZ. So, then they will support those activities. In addition, when we are doing some of the community activities, they also support the communities (KII, government official 13).*

Creating health partnerships extends to supporting the implementation of community-based activities. It was also important to assess which institutions were capable of offering preparatory services to assist with the decentralisation process. For instance, the [general hospital] was identified in the [province] as a training site to train health workers in MDR-TB diagnosis and treatment. For some areas, collaboration with external partners helped them not only train staff members but also led to the rehabilitation of structural facilities that would lead to a smooth decentralisation process of MDR-TB management.

*The [general hospital] is a training and internship site… so we train a lot of interns in MDR TB, of course, our understanding is that as we build capacity, wherever they’ll go, they’ll carry that capacity… we trained pharmacy, trained lab, nurse, clinical people ahh we trained them and trained environmental health for public health purposes (KII, government official 14).*

### Leadership in organising and implementing the training for healthcare workers

The availability of trained human resources for health contributes to their increased knowledge and skills to improve the delivery of TB services. Some healthcare workers reported that after receiving the training, they were now more actively involved in the planning, implementation and monitoring of the delivery of TB services compared with the pre-decentralisation period. However, due to limited funding, several healthcare providers were not trained in the management of MDR-TB.

*So, now we actually started…are equipping, doing capacity building to health workers in these other facilities, which highlights the importance of investing in the development of human resources to improve the overall capacity of the healthcare system* (KII, government official).

### Formation of multidisciplinary teams

The interviewees underscored that creating MDR-TB implementation teams was a crucial step in the decentralisation efforts, at the national, provincial, district, and health facility levels. In this regard, committees and expert teams were formed to spearhead the process. The National Clinical Expert Committee is composed of specialists in internal medicine, and infectious diseases including MDR TB, pharmacy, paediatrics, gynaecology, nutrition, social work and other supporting partners. Collaboration and teamwork were essential for ensuring successful decentralisation efforts, but it was not the same across regions and sites. As one interviewee stated:

*You feel (the patient) is not responding well to treatment, there is a committee that the client is subjected to. They analyse the patient, analyse the drugs, should we switch, should we change maybe from second line treatment…third line treatment. That committee has been there maybe I don’t see any change I don’t think there is something that has changed if there are changes maybe it’s the number of times that probably this committee should sit…the number of times that this committee should look at the patients, discuss the patients…* (KII, government official 14).

### Collaborating with external partners in support decentralisation

For some areas, collaboration with external partners not only helped train health workers but also led to the rehabilitation of existing health facilities’ infrastructure, facilitating the smooth decentralisation process of MDR-TB services and management.

*In 2017, we first started having visitations with NTLP to see what was on the ground… I think the major partner was FHI-360 under the challenge TB program. So, FHI-360 through the challenge TB program conducted the prevention and control training for the entire institution targeting all the workers in all the major departments… and providing infectious control guidelines and activities in each working space in the clinical area as well as in the non-clinical. They brought in partners under USAID and lobbied for us to have an MDR ward rehabilitated. That was done at UTH, here [Kabwe], Ndola and Kitwe, not sure about other provinces if something was done to that effect* (KII, health facility staff 1).

### Joint action in health infrastructural rehabilitation

Furthermore, the collaboration between the Ministry of Health and partners also contributed to improving infrastructure. For some areas, collaboration with external partners helped not only train staff members, but also led to the rehabilitation of structural facilities that would lead to a smooth decentralisation process of MDR-TB management. In some cases, new structures were built for MDR TB management. However, the support was limited as many health facilities required adequate health infrastructure development that remains unmet.

*They brought in partners under USAID and World Bank lobbied for us to have an MDR ward rehabilitated (KII, health facility staff 2).*

## Discussion

This study explored how collaboration influences the effective decentraliation implementation of MDR-TB in Zambia to enhance access and care quality. The principled engagement was shaped by the global health agenda/summit meeting’s influence on the decentralisation of TB, engagement of stakeholders to initiate decentralisation, supportive policy environment and quarterly clinical expert committee meetings. The study underscores the value of collaboration among stakeholders in policy development and implementation, shaping their joint capacity and shared motivation to train healthcare providers and engage communities, ultimately influencing successful treatment outcomes.

The study has revealed that the lack of TB service decentralisation in Zambia led to limited access, hindering eligible patients and clients from conveniently accessing care. However, a Pakistani study showed that expanding the centralised TB healthcare services contributed to increased adverse effects for rural and peri-urban populations [20]. The limited access to TB services in rural and peri-urban areas was attributed to limited or lack of healthcare infrastructure where patients could easily get tested. This highlights the major constraining factors that contributed to limited access to health facilities. They included emergency services transport for referring patients for MDR-TB services, constraining access to health facilities owing to long distances and challenges in providing optimal patient monitoring and review, as motivating factors.

The study suggests that a supportive decentralisation policy and governance environment plays a crucial role in health systems strengthening in MDR-TB in Zambia. The political leadership appreciated the pressing challenges, particularly poor access to MDR-TB services. Therefore, they advocated with political will for a policy shift from centralisation to decentralisation. Similarly, a South African study also showed that the health reform pertaining decentralisation of MDR-TB services was done to enhance access to care by placing TB care closer to communities, and improving TB-care success rates [21]. In addition, studies conducted on health policy and systems reforms also show how critical leadership and power are in driving collective decision-making on health system and policy development and reform [22–24]. The Ministry of Health realised that creating an enabling policy environment would contribute to addressing the limited access to MDR-TB services in Zambia. Therefore, taking services closer to the people promotes equity and contributes to dismantling health inequalities.

The supportive policy health environment spelt out the government’s agenda, direction and commitment to scaling up the decentralisation of MDR-TB services. This roadmap was essential not only in helping health managers, providers and partners understand the policy, but also in giving authority to key stakeholders to hold the government accountable for the status of the delivery of services. An Indian-based study showed that social accountability mechanisms empowered the community to collective negotiations resulting in demands for changes from the health leadership [25]. However, top leadership, in some cases, limited sustained momentum in the decentralisation process. This creates an impression whereby local health actors may fail to appreciate the health reform, contributing to a lack of ownership as they will only be waiting for the superiors to direct the implementation of the process. This study highlights that shared motivation is critical in making the stakeholders understand the programme, facilitate their buy-in and support the creation of the MDR-TB decentralisation structure and plan. Therefore, collaboration is key in facilitating stakeholder engagement through decentralised delivery of TB services to improve accessibility by clients to health facilities and the provision of quality services for a broader population.

Furthermore, this study has highlighted the importance of collaboration in the decentralisation of multi-drug-resistant tuberculosis services. Collaboration plays a crucial role in capacity-building and training among healthcare providers. In South Africa, trained human resources for healthcare are limited, thereby impacting optimal service delivery. Stakeholders, including NGOs’ collaboration and collective action, improved healthcare workers’ delivery of TB services through the provision of specialised healthcare and psychological social support [21, 26, 27]. Furthermore, through joint efforts, healthcare providers can receive specialised training to stay updated with the latest treatment options and management techniques, thus enhancing their proficiency in handling MDR-TB cases.

This study also highlighted that strategic partnerships are essential through capacity-building and training of healthcare providers by contributing to more effective patient care and enhanced treatment outcomes. This finding is in line with other studies, which suggest that collaborative efforts in delivering patient-centred decentralised approaches enable healthcare providers to navigate therapeutic options and provide effective care, ultimately contributing to improved treatment outcomes [4]. Collaboration helps healthcare workers to continue providing services through community structures [28–30]. However, inadequate human resources for health in Zambia is contributing to limiting healthcare provider’s involvement in the treatment of patients. Many healthcare facilities are not fully equipped to handle TB. In addition, they have a limited number of healthcare providers who have heavy workloads with marginal involvement of others in the management of patients.

Some studies have, however, shown that collaboration in delivering a patient-centred decentralised approach where healthcare providers collaborate in delivering TB services helps in navigating therapeutic options and enhances effective care [5]. Furthermore, this study shows that training healthcare providers is key to the decentralisation of TB services. The training equips the officers with specialisation on the latest treatment options in the operations and management of TB. Similarly, evidence from an African study found that equipping healthcare providers in the management of TB and adopting locally appropriate strategies enhances the implementation of the decentralisation policy [31].

Supportive collective community-based MDR-TB interventions were found to be crucial in creating awareness and improving patient treatment outcomes. It was apparent that community health actors, with the involvement of community health workers, contributed to improved awareness, enhanced case detection and strengthened referral systems and monitoring of patients [32]. The findings of the study show that there was inadequate involvement of community-based actors in the delivery of TB services, which might be contributing to low levels of knowledge and inadequate support from the community.

## Limitations and strengths of the study

One of the limitations is the absence of stakeholders from supporting partners, including international organisations. This leaves a gap in understanding engagements during the decentralisation process. This could potentially limit the scope of the insights shaping decentralisation. Another limitation of this study is that we only focussed on collaborative dynamics to understand the key factors shaping the decentralisation policy of MDR-TB services, as it is crucial to provide in-depth knowledge of the key lessons influencing the implementation of these services. Despite this limitation, our study strength includes conducting inclusive interviews with stakeholders at the national, provincial, district and community levels, such as healthcare providers and managers at different levels, patients and caregivers, which facilitated an in-depth understanding of collaboration for implementation of decentralisation policy of multi-drug-resistant tuberculosis services in Zambia. The collaboration of researchers with backgrounds in health, social science and TB programs enhanced the analysis and quality interpretation of the findings.

## Conclusions

The decentralisation of multi-drug-resistant tuberculosis services in Zambia was propelled by collaborative efforts aimed at addressing access to multifaceted challenges arising from the centralised management of TB health services. Collaboration dynamics, including principled engagement, shared motivation and the capacity for joint action, played a crucial role in involving stakeholders to tackle issues such as limited access, transportation barriers and patient monitoring challenges. The shift in policy was grounded in evidence-based decision-making, influenced by political determination and facilitated by supportive policies. However, more capacity-building trainings are needed to increase the number of healthcare workers involved in the delivery of MDR-TB services. The study also identified associated healthcare challenges, including infrastructure and service delivery limitations. Therefore, enhancing stakeholders’ collaboration will create opportunities to expand the current infrastructure and support the optimal decentralised delivery of MDR-TB services.

## Data Availability

The study data can be requested from the authors. The articles for this review can be made available upon request.

## Abbreviations

NGOs: Non-governmental organisations
TB: Tuberculosis
MDR-TB: Multi-drug-resistant tuberculosis
WHO: World Health Organisation
USAID: United States Government via United States Agency for International Development
CDC: Centres for Disease Control and Prevention
JATA: Japan Anti-Tuberculosis Association
CSOs: Civil society organisations
NTLP: National Tuberculosis and Leprosy Programme
UNZABREC: University of Zambia Biomedical Research
